# Anthropophagic Florida mosquito species are poor vectors of prototype and emerging strains of oropouche virus

**DOI:** 10.1371/journal.pntd.0013755

**Published:** 2025-12-01

**Authors:** Dongmin Kim, Tanise Moitinho S. Stenn, Shelby M. Dittman, Yesenia L. Sanchez, Charlotte A. Addae, Limarie J. Reyes Torres, Eva A. Buckner, Nathan D. Burkett-Cadena, Barry W. Alto

**Affiliations:** Department of Entomology and Nematology, Florida Medical Entomology Laboratory, University of Florida, Vero Beach, Florida, United States of America; International Atomic Energy Agency, AUSTRIA

## Abstract

Oropouche virus (OROV) is an emerging vector-borne pathogen in the American tropics that is a significant cause of human disease. Over 100 traveler imported cases of OROV were recorded in the continental USA in 2024 (103 in Florida), elevating the risk of local transmission of OROV should competent insect vectors feed upon viremic humans. The only confirmed natural vector of OROV is *Culicoides paraensis* (Goeldi), a biting midge species that occurs throughout forested areas of the New World, including the eastern USA, Central America and most of northern South America. Anthropophagic mosquito species, especially *Culex quinquefasciatus* Say and *Aedes aegypti* L. have been suspected of transmitting emerging lineages of OROV in areas where *C. paraensis* had not been reported (Cuba). Recent laboratory studies have shown that emerging strains of OROV replicate to higher titers in human cell lines than ancestral strains, raising the possibility that these strains may also be transmitted by select mosquito species that feed on humans. To assess the potential for anthropophagic mosquitoes in the southern USA to transmit OROV, we evaluated the vector competence (based on viral RNA detection) of lab-adapted *Cx. quinquefasciatus* and *Ae. aegypti*, along with an F_1_ generation of *Ae. aegypti* from field-collected mosquitoes in Florida, using prototype (TRVL9760, Trinidad 1955) and emerging (240023, Cuba 2024) OROV genotypes across two incubation periods in cell culture (5 and 7 days) and three extrinsic (7, 14, and 21 days) incubation periods. Both mosquito species exhibited moderate susceptibility to infection (24.2-43.2%) and disseminated infection (23.0-57.5%), but low competence to transmit OROV. Transmission was observed in *Ae. aegypti* (2.5% in the Lower Keys strain and 1.8% in the Orlando strain) and *Cx. quinquefasciatus* (0.7%). When the two viral genotypes (TRVL9760 vs 240023) were compared at the same incubation conditions (IP/EIP), the emergent genotype did not exceed the prototype in infection, dissemination, or transmission. Our data indicates that these two relatively anthropophagic mosquito species are unlikely to serve as competent vectors of OROV in Florida, attributable to substantial midgut and salivary gland barriers.

## Author summary

Oropouche virus (OROV) is an emerging virus that is spreading rapidly across tropical regions of the Americas. In 2024, over 100 travel-related cases were reported in Florida, raising concerns about the potential for local transmission. In South America, OROV is primarily transmitted by a small biting midge called *Culicoides paraensis*. Based on previous findings, mosquitoes are unlikely to play a primary role in OROV transmission. However, it remains unclear whether recently emerged OROV genotypes might interact differently with mosquito populations in Florida, which are widespread and frequently bite humans, under varying environmental conditions. In this study, we tested two common mosquito species in Florida, *Culex quinquefasciatus* and *Aedes aegypti*, to determine whether they could become infected with and transmit a prototype and an emerging genotype of OROV. We found that both species could become infected, but only a small percentage (less than 2.5%) of females were capable of transmission. Our findings provide important data for assessing the risk of local OROV transmission in the southern United States and suggest that these mosquito species are unlikely to serve as efficient vectors.

## Introduction

Oropouche virus (OROV), the causative agent of Oropouche fever, is an emerging vector-borne pathogen of increasing public health concern in the American tropics, where it is recognized as a significant cause of human disease. OROV (*Bunyavirales*: *Peribunyaviridae*, *Orthobunyavirus*) [1] was first isolated in 1955 from the blood of a febrile young male “charcoal burner” who had been working in a forested area on the island of Trinidad [2]. His symptoms including backache, cough, and short-term fever were consistent with other endemic arboviral infections. More broadly, clinical manifestations of OROV infection typically include acute febrile illness, arthralgia, arthritis, and, in severe cases, neurological complications such as Guillain-Barré syndrome, congenital anomalies, and infant mortality [3,4]. Since its initial discovery, OROV has caused sporadic and sometimes widespread outbreaks in the Amazon region of northern Brazil between 1960 and 1989, particularly in the states of Pará and Amazonas, cumulatively resulting in at least 260,000 human infections [5]. Subsequent outbreaks and enhanced surveillance efforts have led to estimates of over 500,000 documented cases in the Americas, though the actual number is likely higher due to underreporting and misdiagnosis [6].

The biting midge *Culicoides paraensis* (Diptera: Ceratopogonidae) is recognized as the primary vector of OROV, based on natural infections in field-collected specimens, experimental transmission studies, and high prevalence of this midge species during outbreaks. OROV has been repeatedly isolated from wild-caught *C. paraensis* during outbreaks in endemic regions of South America, particularly in Brazil [5,7]. Laboratory studies have further confirmed that *C. paraensis* is a competent biological vector, capable of transmitting OROV to vertebrate hosts such as hamsters following oral infection [8]. Pinheiro et al. (1982) demonstrated that *C. paraensis* became infected after feeding on viremic human patients, with an overall infection rate of 15% (27/181) across all bloodmeal exposures. Among the infected midges, 44% (12/27) successfully transmitted OROV to hamsters. Additionally, the spatial and temporal distribution of *C. paraensis* closely corresponds with reported outbreaks of Oropouche fever in Brazil and Peru [9].

In addition to *C. paraensis*, several mosquito species such as *Coquillettidia venezuelensis*, *Aedes serratus*, and *Culex quinquefasciatus* were initially suspected as potential vectors of OROV based on virus isolations from field-collected specimens, their widespread distribution, and their established roles in the transmission of other arboviruses [2,3,5,7,10,11]. Comprehensive field and laboratory investigations suggest that the role of these mosquito species in OROV transmission is likely limited, given the low number of OROV-positive specimens collected in the field and their low vector competence, even when exposed to high viral titers [10,12–14].

The implicated natural vertebrate hosts of OROV include the three-toed sloth (*Bradypus tridactylus*), which was found to be infected with OROV [7] and non-human primates (e.g., *Callithrix* spp.), which produce substantial viremia when inoculated with OROV [3]. OROV antibodies have been detected in a variety of wild mammals and birds, including non-human primates (e.g., *Callithrix* spp.), rodents (*Proechimys* spp.), and marsupials (*Caluromys philander*) [3,15,16]. These findings suggest that a diverse range of vertebrate hosts may be exposed to OROV, potentially facilitating spillover events into human populations in endemic areas [17].

While much of the early work focused on endemic transmission dynamics, more recent events highlight OROV’s geographic expansion. A notable example is the emergence of OROV in the Caribbean, particularly the large outbreak in Cuba in 2024, which resulted in over 500 confirmed human cases of Oropouche fever occurring all 15 provinces. At the time of the outbreak *C. paraensis* had not been recorded from Cuba, nor had natural vertebrate hosts, *B. tridactylus* [18], and viral reassortment, generating strains that replicate to higher titers in human cells over shorter periods compared to ancestral strains was suspected to drive expansion of OROV [19]. For instance, the 2023–2024 epidemic strain AM0088 from Brazil produced higher extracellular titers than the historical prototype strain BeAn19991 in monkey (Vero CCL81) and human (Huh7 and U251) cell lines [19]. In addition, OROV possesses a tripartite genome (L, M, and S segments), which promotes diversification through both reassortment and mutation. This genomic flexibility can enhance the virus’s adaptability, expands its host range, and contributes to its potential for geographic expansion [20]. Genetic changes may also alter virus–vector interactions that influence vector competence, as demonstrated in previous studies of chikungunya virus and West Nile virus [21,22]. These findings underscore the importance of reassessing vector competence under changing ecological and virological conditions that may facilitate novel transmission pathways.

This need is further supported by recent detections of imported OROV cases in non-endemic regions, including the United States. In 2024, a notably high number of imported cases were reported in Florida (n = 103), at least ten times more than any other U.S. state [23]. The presence of potential vectors and suitable environmental conditions in subtropical and temperate regions raises concerns about the possibility of local transmission in the United States. Evaluating the vector competence of locally abundant mosquito species is essential for anticipating the risk of local OROV transmission, particularly if the virus becomes established and begins circulating within vector and vertebrate populations.

To better understand the potential for local OROV transmission in the U.S., we evaluated the vector competence of laboratory-adapted strains of *Cx. quinquefasciatus* Say and *Aedes aegypti* L. as well as a recently established *Ae. aegypti* colony (F_1_ generation from field-collected mosquitoes), for both the prototype (TRVL9760, 1955) and emergent (240023, 2024) strains of OROV. We also assessed vector competence across different incubation periods in cell culture and extrinsic incubation periods, which are critical factors influenced by ecological and virological conditions that can significantly affect transmission dynamics. The incubation period in cell culture (IP) refers to the duration the virus was allowed to replicate in Vero cells prior to mosquito exposure. The extrinsic incubation period (EIP) refers to the time between a mosquito acquiring the virus during blood feeding and when the virus reaches the saliva, enabling transmission to a new host. Given the high OROV viremia observed in humans [3,8,24,25] and the absence of the primary enzootic host, the three-toed sloth (*B. tridactylus*), in Florida, evaluating the vector competence of anthropophagic mosquito species is essential to determine whether these widespread species should be targeted for control in response to future travel-associated cases.

## Methods

### Mosquitoes

Female mosquitoes of *Cx. quinquefasciatus* (Vero Beach–Gainesville strain, established in 2015) and *Ae. aegypti* (Orlando strain, 1952 and Lower Keys strain, 2024) used in transmission experiments were reared from colonies established from Florida populations of the two species. *Aedes aegypti* (Lower Keys strain) used in this study were F₁ progeny derived from field-collected mosquitoes obtained in 2024 from the Lower Florida Keys (GPS coordinates: 24.557722, –81.779025). Larvae were reared under low-density conditions to promote optimal size and condition of emerging adults [26]. In brief, eggs (rafts or preserved eggs) were hatched in plastic pans with tap water and suspended larval food [27]. First instar larvae were manually thinned to a density of approximately 100 larvae per liter [28]. Larvae were provided with a 1:1 mixture of yeast and lactalbumin every other day [29]. Pupae were manually transferred to containers with clean tap water for emergence in cages supplied with 10% sucrose solution *ad libitum* via moistened cotton pledgets. Adult females used in experiments were 4–7 days post emergence. Prior to vector competence evaluation, mosquitoes were starved (no sucrose water) for 18 hr.

### OROV infection

**Fig 1:**
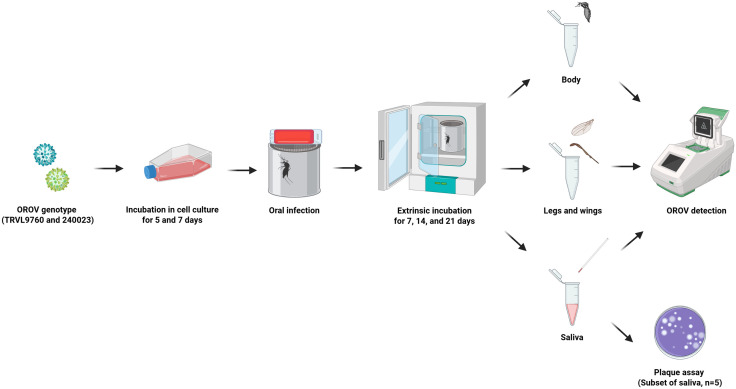
Overview of the Experimental Workflow. Oropouche virus infection, dissemination, and transmission were evaluated across various genotypes, incubation periods in cell culture, and extrinsic incubation periods. Illustration figures were created with biorender.com.

An overview of the experimental workflow is presented in Fig 1. Two genotypes of Oropouche virus (OROV TRVL9760 isolated from a health unit in Grande, Trinidad, West Indies, in 1955 and OROV 240023 isolated from a traveler to Cuba in the USA in 2024) were obtained from the Centers for Disease Control and Prevention. Each OROV genotype was propagated in tissue culture flasks (175 cm²) containing confluent monolayers of Vero cells (Vero E6, ATCC CRL-1586). For each OROV genotype, cell monolayers were inoculated at a multiplicity of infection of 0.01 virions per cell. After 45 minutes of incubation at 37°C in a 5% CO₂ atmosphere, 25 mL of media consisting of 199 media, 10% fetal bovine serum, 0.2% amphotericin B (Fungizone™), and 2% penicillin-streptomycin was added to the flasks, following previously established methods [27]. The tissue culture flasks containing the virus were harvested after five and seven days of incubation at 37°C with 5% CO₂, representing two distinct virus incubation periods on Vero cells. After each incubation period, the virus-containing supernatant was harvested and immediately used (i.e., without freezing) to prepare infectious blood meals for mosquito feeding. Bloodmeal titers for each incubation period were determined separately.

**Fig 2:**
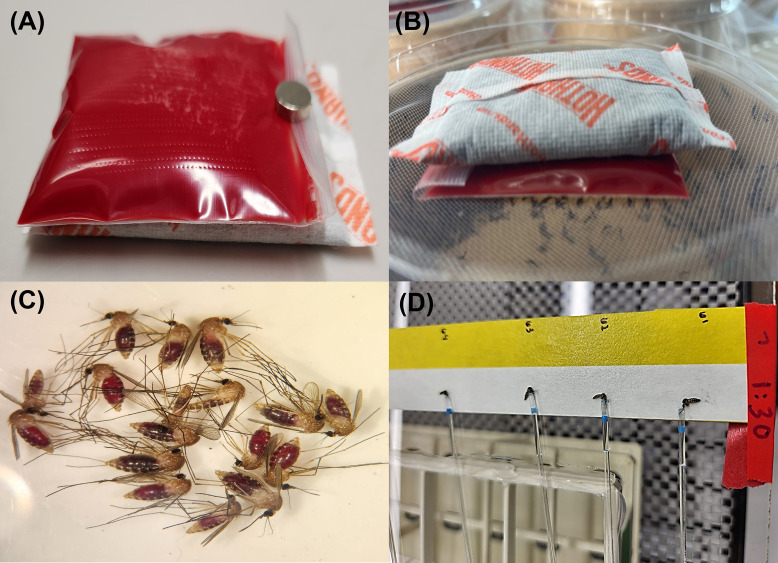
(A) Perforated bags containing Oropouche virus (OROV) suspension and defibrinated bovine blood placed on hand warmers. (B) Perforated bag positioned atop a cylindrical cardboard mosquito cage for blood feeding. (C) Blood-engorged *Culex quinquefasciatus* females. (D) Immobilized female mosquitoes prepared for saliva collection using capillary tubes filled with immersion oil. Photo credit: Nathan Burkett-Cadena. Used with permission and published under the Creative Commons Attribution 4.0 International (CC BY 4.0) license.

A novel plastic pouch and hand-warmer method was developed to provide feeding mosquitoes with a blood–virus suspension (Fig 2). In brief, a small resealable sliding-channel storage bag (5.08 × 7.62 cm; RKZCT, Amazon Products, WA, USA) was prepared by placing a 4.0 × 4.0 cm piece of cellulose fiber and cotton sponge inside and puncturing one side with a 0.25 mm microneedle derma roller (Sdara Skincare, CA, USA) to create ~200–300 perforations. The bag was then filled with 20 mL of a 1:1 mixture of defibrinated bovine blood and OROV suspension, gently compressed to remove air, and sealed with 15 mm × 2.0 mm magnets (TRYMAG, Amazon Products, WA, USA) to prevent leakage (Fig 2A). Five minutes before feeding, disposable hand warmers (Kobayashi Americas, GA, USA) were activated and placed on top of the perforated bag to maintain the blood at vertebrate body temperature (~37°C). The bag and warmer were positioned on the mesh screen lids of shallow, bowl-shaped cardboard cages (Ø 16.51 cm top, Ø 14.48 cm bottom, height 6.60 cm; SINJEUN, Amazon Products, WA, USA) for 60 minutes (Fig 2B). After feeding, a 1.5 mL aliquot of the blood–virus suspension was collected and stored at –80°C for subsequent plaque assay titration to verify the infectious dose provided to mosquitoes.

After feeding trials, female mosquitoes were immobilized by placing cages in a cooler with wet ice for 10–15 minutes (Fig 2C). Only blood-engorged females were selected based on confirmed feeding status and transferred into cardboard mosquito cages. The cages were maintained for extrinsic incubation periods of seven, fourteen, and twenty-one days in incubators set at 27.0 ± 0.5°C, 80.0 ± 5.0% relative humidity, and a 14:10 (L:D) h photoperiod. After the designated incubation period, females that had fed on the blood-virus suspension were cold-anesthetized and transferred onto enamel-coated metal pans placed on wet ice within a biosecurity glove box. Mosquitoes were dissected with sterilized forceps to detach legs and wings, which were reserved for disseminated infection assays. Immobilized bodies (head, thorax, abdomen) were then placed on double-sided tape, and their proboscis was inserted into capillary tubes filled with immersion oil for 45 minutes to collect saliva, as previously described [30]. The immersion oil was transferred into 1.5 mL microcentrifuge tubes containing 300 µL of Medium 199 (M199) (Fig 2D). After saliva collection, head–thorax–abdomen samples were placed into 2 mL microcentrifuge tubes containing 1 mL of M199, and legs and wings were placed into separate 2 mL tubes containing 1 mL of the same medium. Two sterilized stainless-steel ball bearings (BBs) were added to each tube, and samples were homogenized in a TissueLyser II sample disruptor (Qiagen, MD, USA) at 19.5 Hz for 3 minutes. All samples were stored at −80°C until virus quantification by RT-qPCR. A subset of samples, including virus stocks, infectious blood meals, and RT-qPCR-positive saliva samples, was also tested by plaque assay.

### RT-qPCR assays

Viral RNA was extracted from the samples using the QIAamp Viral RNA Mini Kit (Qiagen, CA, USA) according to the manufacturer’s instructions. RT-qPCR amplification was performed using the SuperScript III Platinum One-Step RT-qPCR Kit (Thermo Fisher Scientific, MA, USA) on a CFX96 Real-Time PCR Detection System (Bio-Rad Laboratories, CA, USA). The cycling conditions consisted of reverse transcription at 50°C for 15 minutes, followed by enzyme inactivation and initial denaturation at 95°C for 2 minutes, and 45 cycles of 95°C for 15 seconds and 60°C for 30 seconds. Each 20 μL reaction contained 10 µL of 2x reaction buffer (Invitrogen, CA, USA), 0.4 μL of SuperScript™ III One-Step RT-PCR enzyme mix (Invitrogen, CA, USA), 1.2 μL of forward primer (10 μM), 1.2 μL of reverse primer (10 μM), 0.4 μL of probe (10 μM), 1.8 μL of DEPC-treated water (Fisher BioReagents, PA, USA), and 5 μL of RNA template. The assay targeted the S segment of the Oropouche virus (OROV) genome using primers and a probe adapted from Naveca et al. [31]. 

The sequences were: forward primer, 5′–TCCGGAGGCAGCATATGTG–3′; reverse primer, 5′ACAACACC AGCATTGAGCACTT–3′; and probe, 5′–FAM CATTTGAAGCTAGATACGG–NFQ–MGB–3′. Standard curves were generated using ten-fold serial dilutions of quantified RNA from both genotypes of OROV. The assay reliably detected low concentrations of viral RNA, with consistent amplification observed at the lowest plaque-titrated dilutions included in the standard curves for both genotypes (see S1 Fig). No amplification was observed in no-template controls. Samples with Cq values ≤38 were considered positive, following CDC interpretation criteria for OROV RT-qPCR using the SuperScript III platform. Because in-vitro–transcribed RNA standards were not used, absolute genome copy numbers were not calculated. Instead, Cq values were converted to PFU/mL equivalents using standard curves generated from serial dilutions of plaque-titrated OROV genotypes. These curves indicated empirical limits of detection of approximately 0.3 PFU/mL for the TRVL9760 genotype and 0.08 PFU/mL for the 240023 genotype, demonstrating comparable assay sensitivity across genotypes and confirming that both assays detect virus well below 1 PFU/mL.

### Plaque assay

Virus titers from suspensions of two genotypes (TRVL9760 and 240023) at two incubation periods in cell culture (five and seven days) were quantified by plaque assays on Vero cells, both before and after mixing with bovine blood. Additionally, saliva samples from mosquitoes with confirmed viral dissemination (i.e., RT-qPCR–positive leg and wing samples; N = 339) were screened by RT-qPCR, and five were found to be positive, as shown in Table 1. These five RT-qPCR–positive saliva samples were subsequently tested by plaque assay to quantify viral titer and assess the presence of infectious virus. Each stock or blood-virus suspension was serially diluted 1:10 (from 10 ⁻ ¹ to 10 ⁻ ⁹) and inoculated onto Vero cell monolayers seeded in 12-well plates (n = 3 technical replicates per treatment). Monolayers were seeded at a density of 1.2 × 10⁵ cells/mL and inoculated with 200 µL of the sample plus 200 µL of complete media, followed by 1 hour of incubation at 37°C with 5% CO₂. After incubation, each well received 2 mL of 1% methylcellulose overlay prepared in DMEM containing 5% EquaFetal, 1% PenStrep, and 1% L-glutamine, and plates were incubated for three days. Following incubation, the media and agarose overlay were removed, and the plates were stained with 0.25% crystal violet after fixation with 10% formalin and 1% methylene blue. Plates were then rinsed with tap water, and visible plaques were counted under a light illuminator. Each plaque was assumed to have originated from a single viral infection.

**Table 1:** Mean infection, dissemination, and transmission rates (conditional rates) for *Culex quinquefasciatus* (Vero Beach strain, 2015) and *Aedes aegypti* (Orlando strain, 1952; Lower Keys strain, 2024) after exposure to two Oropouche virus (OROV) genotypes (TRVL9760 and 240023). Virus suspensions were harvested after 5 or 7 days of incubation in Vero cell culture, corresponding to higher or lower bloodmeal titers, respectively (incubation period in cell culture, IP; see footnote for titers). The Lower Keys strain represents F_1_ progeny from field-collected *Ae. aegypti* in 2024. Rates were evaluated at 7-, 14-, and 21-day extrinsic incubation periods (EIP). Infection rates were calculated as the number of mosquitoes with RT-qPCR–positive bodies divided by the total number blood-fed. Dissemination rates were calculated as the number with RT-qPCR–positive legs/wings divided by the number of body-positive mosquitoes. Transmission rates were calculated as the number with RT-qPCR–positive saliva divided by the number of dissemination-positive mosquitoes. All samples were tested for OROV RNA using RT-qPCR, with samples considered positive if Cq ≤ 38. Detection of viral RNA does not confirm the presence of infectious virus. A dash (–) indicates that samples were not collected or not tested under that condition.

OROV Genotype	Mosquito Species	Strain	Sample Type	5 IP (Higher OROV titer)*			7 IP (Lower OROV titer)*		
				7 EIP	14 EIP	21 EIP	7 EIP	14 EIP	21 EIP
TRVL9760	*Cx. quinquefasciatus*	Vero Beach	Infection (% [n/N])	26.1% (12/46)	30.4% (14/46)	15.2% (7/46)	17.4% (8/46)	10.9% (5/46)	14.6% (7/48)
			Dissemination (% [n/N])	75.0% (9/12)	7.1% (1/14)	0.0% (0/7)	37.5% (3/8)	0.0% (0/5)	28.6% (2/7)
			Transmission (% [n/N])	0.0% (0/12)	0.0% (0/14)	0.0% (0/7)	0.0% (0/8)	0.0% (0/5)	0.0% (0/7)
	*Ae. aegypti*	Orlando	Infection (% [n/N])	21.7% (10/46)	60.9% (28/46)	19.6% (9/46)	40.0% (16/40)	4.3% (2/46)	4.2% (2/48)
			Dissemination (% [n/N])	0.0% (0/10)	14.3% (4/28)	0.0% (0/9)	25.0% (4/16)	0.0% (0/2)	50.0% (1/2)
			Transmission (% [n/N])	0.0% (0/10)	0.0% (0/28)	0.0% (0/9)	6.3% (1/16)	0.0% (0/2)	0.0% (0/2)
	*Ae. aegypti*	Lower Keys	Infection (% [n/N])	–	66.7% (8/12)	–	–	52.9% (9/17)	–
			Dissemination (% [n/N])	–	87.5% (7/8)	–	–	55.6% (5/9)	–
			Transmission (% [n/N])	–	12.5% (1/8)	–	–	0.0% (0/9)	–
240023	*Cx. quinquefasciatus*	Vero Beach	Infection (% [n/N])	19.6% (9/46)	23.9% (11/46)	80.4% (37/46)	26.1% (12/46)	15.2% (7/46)	10.9% (5/46)
			Dissemination (% [n/N])	66.7% (6/9)	0.0% (0/11)	16.2% (6/37)	33.3% (4/12)	0.0% (0/7)	20.0% (1/5)
			Transmission (% [n/N])	0.0% (0/9)	9.1% (1/11)	0.0% (0/37)	0.0% (0/12)	0.0% (0/7)	0.0% (0/5)
	*Ae. aegypti*	Orlando	Infection (% [n/N])	60.9% (28/46)	21.7% (10/46)	19.6% (9/46)	34.8% (16/46)	12.5% (6/48)	63.0% (29/46)
			Dissemination (% [n/N])	57.1% (16/28)	40.0% (4/10)	0.0% (0/9)	12.5% (2/16)	16.7% (1/6)	20.7% (6/29)
			Transmission (% [n/N])	3.6% (1/28)	0.0% (0/10)	0.0% (0/9)	6.3% (1/16)	0.0% (0/6)	0.0% (0/29)
	*Ae. aegypti*	Lower Keys	Infection (% [n/N])	–	37.0% (17/46)	–	–	35.0% (7/20)	–
			Dissemination (% [n/N])	–	62.5% (10/16)	–	–	14.3% (1/7)	–
			Transmission (% [n/N])	–	0.0% (0/16)	–	–	0.0% (0/7)	–

*Titers of OROV blood-virus suspensions, determined by plaque assay, were: TRVL9760 (5-day IP), 6.27 × 10⁵ PFU/mL; TRVL9760 (7-day), 2.73 × 10⁵; 240023 (5-day), 4.67 × 10⁵; and 240023 (7-day), 1.11 × 10⁵ PFU/mL.

### Data analysis

Infection, dissemination, and transmission rates were calculated using both total and conditional approaches. Total rates were defined as the percentage of mosquitoes with OROV-positive bodies, legs/wings, or saliva out of all individuals tested. Conditional rates were calculated as follows: dissemination = leg/wing-positive ÷ body-positive; transmission = saliva-positive ÷ dissemination-positive. Saliva from dissemination-positive mosquitoes was screened by RT-qPCR, and RT-qPCR–positive samples were further tested by plaque assay to assess viral titer and confirm the presence of infectious virus. Comparisons of OROV titers across incubation periods in cell culture and genotypes were performed using one-way ANOVA (SAS Institute Inc., Cary, NC, USA). Logistic regression analyses were used to determine whether categorical variables such as viral genotype, incubation period in cell culture (IP, used as a proxy for higher vs. lower bloodmeal titers), and extrinsic incubation period (EIP) significantly influenced OROV positivity across different mosquito species (or strains). The effects of variables and their interactions on OROV positivity in infection, dissemination, and transmission were evaluated using Type III likelihood ratio test (LRT). A *p*-value of less than 0.05 was considered statistically significant.

## Results

### OROV standard curves and viral titers in stock and blood-virus suspensions

**Fig 3:**
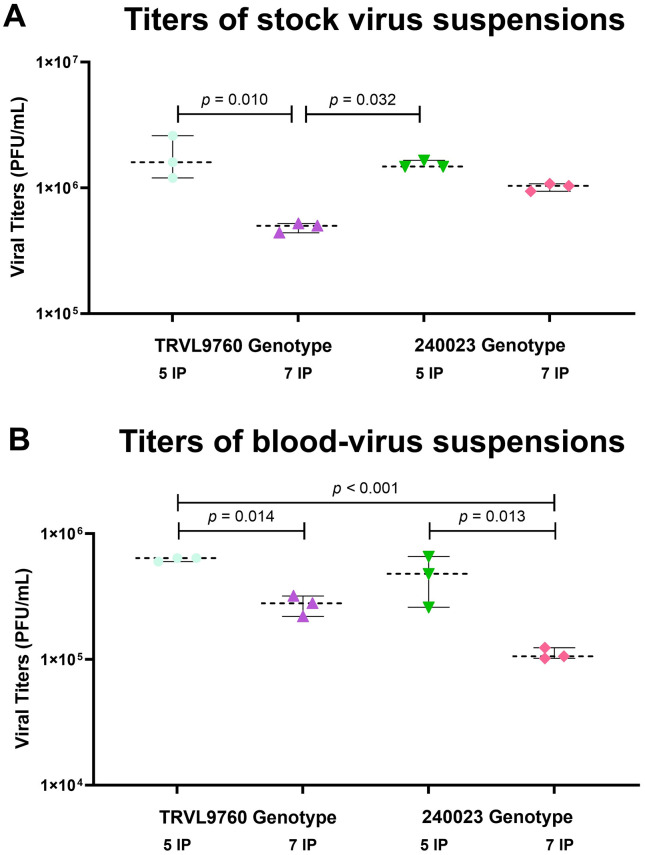
Titers of (A) stock virus suspensions and (B) blood-virus suspensions used for mosquito oral infection, containing two Oropouche virus (OROV) genotypes (TRVL9760 and 240023) incubated for 5 or 7 days in Vero cell culture. Titers were quantified by plaque assay. Data points represent technical replicates; horizontal lines indicate the median and 95% confidence intervals. Statistical significance was assessed using one-way ANOVA followed by Tukey’s HSD test. Exact p-values for significant pairwise comparisons are shown (p < 0.05).

Standard curves for OROV genotypes TRVL9760 and 240023 are presented in S1 Fig. Virus suspensions obtained after five days of incubation in Vero cell culture exhibited higher titers than those from seven days, regardless of genotype (Fig 3A). For example, TRVL9760 suspensions at five days reached 1.80 × 10⁶ PFU/mL, significantly exceeding titers at seven days (4.87 × 10⁵ PFU/mL; *p* = 0.010). Because blood-virus suspensions were prepared as 1:1 dilutions of these stocks, bloodmeal titers similarly reflected the higher titers of five-day stocks compared to seven-day stocks for both virus genotypes (Fig 3B).

### Variation in infection, dissemination, and transmission rates

A total of 1,877 samples were processed for OROV detection, including *Cx. quinquefasciatus* (Vero Beach strain, n = 822), *Ae. aegypti* (Orlando strain, n = 880), and *Ae. aegypti* (Lower Keys strain, n = 175), as summarized in Table 1. Infection rates varied substantially by mosquito species (or strain), viral genotype, and incubation period (IP), with 5-day incubations corresponding to higher bloodmeal titers and 7-day incubations to lower titers. The highest infection rate (80.4%) was observed in *Cx. quinquefasciatus* (Vero Beach strain) fed on blood containing genotype 240023 virus harvested at 5 days (higher titer), with infection assessed after a 21-day extrinsic incubation period. Because dissemination and transmission were assessed only in mosquitoes with confirmed infections, the sample sizes for these outcomes varied accordingly. To reflect the overall transmission potential and facilitate comparisons across studies, total infection, dissemination, and transmission rates based on the total number of mosquitoes tested are provided in S1 Table. The highest dissemination rate (87.5%) was observed in *Ae. aegypti* (Lower Keys strain) exposed to genotype TRVL9760 harvested at 5 days (higher titer), assessed after a 14-day extrinsic incubation period. Transmission rates were generally low across all species (or strains) and conditions, with the highest value (12.5%) also recorded in *Ae. aegypti* (Lower Keys strain) under the same condition.

### RT-qPCR–based infection rates

**Fig 4:**
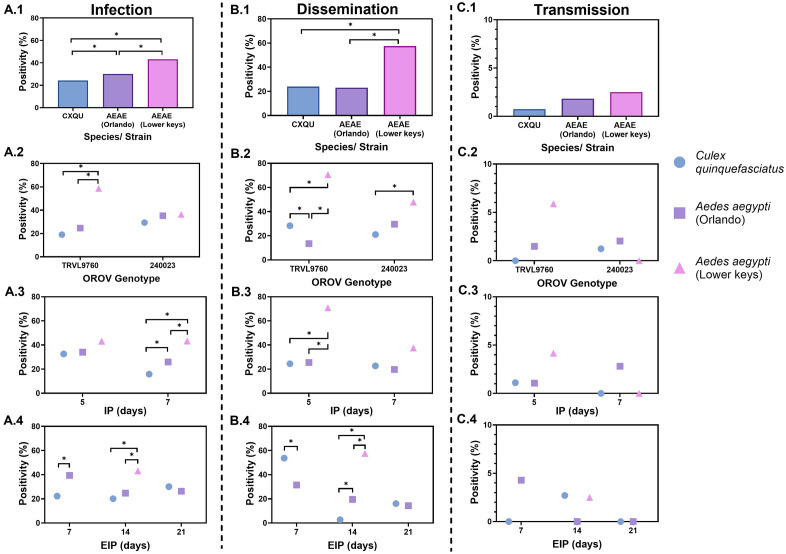
Mean (A) infection (head, thorax, and abdomen), (B) dissemination (legs and wings), and (C) transmission (saliva) rates of *Culex quinquefasciatus* (CXQU, Vero Beach strain) and *Aedes aegypti* (AEAE, Orlando and Lower Keys strains) infected with Oropouche virus (OROV) genotypes TRVL9760 and 240023. Positivity rates were calculated as follows: infection = positive bodies/ total mosquitoes tested; dissemination = positive legs/ infected bodies; transmission = positive saliva/ mosquitoes with disseminated virus. Data points represent sample-size–weighted mean positivity (%) calculated from individual mosquito-level binomial outcomes. Wilson score 95% confidence intervals for all infection, dissemination, and transmission rates are provided in S4 Table. Asterisks (*) indicate statistically significant differences among mosquito strains at each variable level, based on logistic regression with likelihood ratio tests (p < 0.05). Panel labels (A1–C4) correspond to comparisons across (1) strain, (2) genotype, (3) incubation period in cell culture, and (4) extrinsic incubation period. Marker shape and color indicate mosquito strain: blue circle = CXQU, purple square = AEAE Orlando, and pink triangle = AEAE Lower Keys. Missing data points reflect treatment combinations not tested due to mosquito availability constraints.

Mosquito species (or strains) significantly influenced OROV infection outcomes (χ² = 16.22, *p* = 0.0005) (Fig 4A; rates shown were calculated as pooled, sample-size–weighted binomial proportions [total positives ÷ total tested]). Overall, the *Ae. aegypti* (Lower Keys strain) exhibited the highest body infection rate (43.2%), followed by the Orlando strain (30.0%) and *Cx. quinquefasciatus* (24.2%). When stratified by OROV genotype, the Lower Keys strain of *Ae. aegypti* showed the highest body infection rate (36.4%) in response to the TRVL9760 genotype, compared to the Orlando strain of *Ae. aegypti* (35.3%) and *Cx. quinquefasciatus* (29.3%) (χ² = 19.70, *p* < 0.0001). In contrast, no significant differences in body infection rates were observed among mosquito populations exposed to the 240023 genotype (χ² = 2.64, *p* = 0.2667). Analysis by incubation period in cell culture (IP) showed no significant difference in body infection rates at 5 days IP (χ² = 2.29, *p* = 0.3186). However, at 7 days IP, the Lower Keys strain of *Ae. aegypti* exhibited the highest infection rate (43.1%), significantly greater than those of *Cx. quinquefasciatus* (32.6%) and the Orlando strain (34.1%) (χ² = 17.27, *p* = 0.0002). Similarly, at 14 days extrinsic incubation period (EIP), the Lower Keys strain of *Ae. aegypti* maintained the highest body infection rate (43.1%), which was significantly higher than those observed in *Cx. quinquefasciatus* (20.1%) and the Orlando strain of *Ae. aegypti* (24.7%) (χ² = 16.62, *p* = 0.0002). Logistic regression detected a significant interaction effect on OROV susceptibility to infection (S2 Table). Infection rates were significantly affected by mosquito species (or strains), OROV genotype, and both incubation periods (IP and EIP). A significant four-way interaction suggested that infection outcomes were influenced by a combination of all these factors. To further characterize infection intensity, we examined the distribution of Cq values among RT-qPCR positive mosquito bodies (S3 Table). Because certain treatment groups included very few RT-qPCR–positive mosquitoes (see S3 Table), the associated standard deviation (SD) values are larger in those groups, reflecting small-sample variability rather than greater biological dispersion. Across all groups, Cq values ranged from 15.3 to 37.9. Notably, lower Cq values, indicating higher viral RNA loads, were observed in *Ae. aegypti* (Lower Keys strain) exposed to the 240023 genotype, particularly at 14 days EIP. In contrast, higher Cq values approaching the assay’s detection limit were more frequently detected in *Cx. quinquefasciatus* and in mosquitoes exposed to the TRVL9760 genotype, especially at 7 days EIP.

### RT-qPCR–based dissemination rates

Significant differences in OROV dissemination rates were observed among mosquito species (or strains) (χ² = 29.52, *p* < 0.0001). *Aedes aegypti* (Lower Keys strain) exhibited the highest overall dissemination rate (57.5%), followed by the Orlando strain (23.0%) and *Cx. quinquefasciatus* (23.9%) (Fig 4B). When exposed to the TRVL9760 genotype, the Lower Keys strain of *Ae. aegypti* showed the highest dissemination rate (47.8%), compared to 29.6% in the Orlando strain and 21.0% in *Cx. quinquefasciatus* (χ² = 21.19, *p* < 0.0001). For genotype 240023, a significant difference in dissemination was only detected between the Lower Keys strain of *Ae. aegypti* and *Cx. quinquefasciatus* (*p* = 0.0137) with the former species exhibiting higher disseminated infection. Dissemination patterns across incubation period in cell cultures (IP) revealed significant variation at 5 days (χ² = 19.24, *p* < 0.0001), with dissemination peaking in the Lower Keys strain of *Ae. aegypti* (70.8%), and substantially lower rates observed in the Orlando strain (25.5%) and *Cx. quinquefasciatus* (24.4%). By 7 days IP, dissemination declined across all groups: 37.5% in the Lower Keys strain of *Ae. aegypti*, 19.7% in the Orlando strain, and 22.2% in *Cx. quinquefasciatus* (χ² = 2.14, *p* = 0.3427), a pattern consistent with trends observed in body infection rates (Fig 4A). Dissemination rates also varied by extrinsic incubation period (EIP). At 14 days EIP, the Lower Keys strain of *Ae. aegypti* maintained the highest dissemination (57.5%), which was significantly greater than rates in the Orlando strain (19.6%) and *Cx. quinquefasciatus* (2.7%) (χ² = 33.80, *p* < 0.0001). A significant difference was also noted between the Orlando strain of *Ae. aegypti* and *Cx. quinquefasciatus* at 7 days EIP (*p* = 0.0212). No significant differences in dissemination were observed among mosquito populations at 21 days EIP. Logistic regression identified a significant interaction effect on OROV dissemination rates (S2 Table). Dissemination was significantly influenced by mosquito species (or strains), IP, and EIP, with additional effects from the interaction between OROV genotype and IP. A significant interaction between species (or strains) and EIP, along with a three-way interaction (species/strain × IP × EIP), indicated that dissemination dynamics varied depending on both mosquito and temporal factors. Across all groups, Cq values ranged from 18.1 to 37.9 and generally increased with extrinsic incubation period, with means of 33.8 ± 4.0 at 7 days, 34.8 ± 3.3 at 14 days, and 35.4 ± 2.5 at 21 days, indicating a decline in viral RNA load over time. Lower Cq values were most frequently observed in *Ae. aegypti* (Lower Keys and Orlando strains) infected with the 240023 genotype, whereas higher values were more common in *Cx. quinquefasciatus* and in mosquitoes exposed to the TRVL9760 genotype.

### RT-qPCR–based transmission rates

Transmission rates did not significantly differ among mosquito species (or strains) (χ² = 0.58, *p* = 0.7483). *Aedes aegypti* (Lower Keys strain) exhibited the highest average transmission rate (2.5%), followed by the Orlando strain (1.8%) and *Cx. quinquefasciatus* (0.7%) (Fig 4C). Although these values indicate a trend toward higher transmission potential in the Lower Keys strain, the observed differences were not statistically significant. Additionally, no significant variation in transmission was detected when stratified by viral genotype, incubation period in cell culture (IP), or extrinsic incubation period (EIP), indicating consistent transmission potential across mosquito populations regardless of these factors. Logistic regression (S2 Table) revealed significant three-way interactions, indicating that transmission rates varied depending on the combination of mosquito species (or strains), OROV genotype, and incubation period. *Aedes aegypti* inoculated with OROV 240023 (5-day incubation period) and held for shorter extrinsic incubation periods (7 and 14 days) tended to exhibit higher transmission rates. The lowest Cq (27.9) was found in *Ae. aegypti* (Lower Keys strain) exposed to the TRVL9760 genotype at 14 days EIP. Higher Cq values were observed in *Ae. aegypti* Orlando strain and *Cx. quinquefasciatus* exposed to the 240023 genotype, especially at 7 and 14 days EIP.

### Detection of infectious OROV in saliva by plaque assay

Of the five mosquito saliva samples that tested positive for OROV RNA by RT-qPCR (Cq values: 38.0, 37.7, 35.6, 27.9, and 36.9; S5 Table), only two yielded quantifiable plaques in Vero cells: 5 PFU/mL from *Cx. quinquefasciatus* (Vero Beach strain, 240023 genotype; 5 IP and 14 EIP; Cq = 37.7) and 25 PFU/mL from *Ae. aegypti* (Orlando strain, TRVL9760 genotype; 7 IP and 7 EIP; Cq = 36.9). The remaining RT-qPCR positive saliva samples, *Ae. aegypti* (Orlando, 240023, 7 IP and 7 EIP, Cq = 38.0), *Ae. aegypti* (Orlando, 240023, 5 IP and 7 EIP, Cq = 35.6), and *Ae. aegypti* (Lower Keys, TRVL9760, 5 IP and 14 EIP, Cq = 27.9), were negative by plaque assay. Because saliva volume was limited, plaque assays could not be repeated for all samples, and in some cases only a single plaque was observed; therefore, titers are reported only when plaques were clearly distinguishable and reproducible.

## Discussion

Our laboratory results demonstrate overall low vector competence for Florida-derived strains of the anthropophagic mosquito species, *Ae. aegypti* and *Cx. quinquefasciatus*. Both species exhibited moderate overall susceptibility to OROV infection (24.2-43.2%), and disseminated infection (23.0-57.5%), however very few females with disseminated infections were found to be capable of transmitting OROV (0.7-2.5%), as determined by presence of OROV RNA in saliva. These findings are consistent with results of previous studies that have evaluated the vector competence of other anthropophagic mosquito species in the USA [14,32] and Brazil [12,33]. To our knowledge, just one other study [14] has compared the vector competence of anthropophagic mosquito species for prototype (OROV TRVL9760) and emerging (OROV 240023) strains of OROV virus. In that study, infection rates in *Cx. quinquefasciatus* were just 2% of 96 females screened, which was substantially lower than body infection rates observed in our work. Body infection rates in other mosquito species (*Aedes albopictus, Anopheles quadrimaculatus,* and *Culex pipiens*) evaluated by Payne et al. [14] were similarly low (0–4%). While the body and disseminated infection rates in *Cx. quinquefasciatus* and *Ae. aegypti* were generally higher in our study than those observed for the same species in previous studies [13], these values reflect detection of viral RNA by RT-qPCR rather than infectious virus, which likely accounts for the observed differences. Notably, these increases did not translate into higher transmission. Within the tested OROV bloodmeal titer range (~1.1 × 10^5^–6.3 × 10^5^ PFU/mL; plaque assay), infection rates did not increase with higher titer. Interestingly, transmission rates declined with longer extrinsic incubation periods, which contrasts with the typical trend of increasing transmission over time. This may reflect stochastic variation due to the small number of saliva-positive samples or virus clearance during extended incubation. Previous arbovirus infection studies have identified temporal changes in viral titer among mosquito organs (*Ae. aegypti* infected with dengue-2 virus) [34,35], or a decline in mosquito transmission efficacy following peak transmission during the EIP (*Ae. aegypti* and *Ae. albopictus* infected with chikungunya virus) [36,37]. These researchers suggested that modulation of viral titers in different organs, attributable to mosquito or viral factors, as being responsible for temporal changes in viral titer in mosquito organs. For example, the mosquito’s RNA interference (RNAi) pathway has been shown to inhibit virus replication mediated by small interfering RNAs (siRNAs) and modulate dengue virus infection in *Ae. aegypti* [35]. In the current study, although based on limited data, this pattern warrants further investigation of OROV transmission dynamics to identify the mechanism(s) responsible for modulation of OROV infection.

The highest overall infection, disseminated infection, and transmission rates observed in our study were from the F_1_ progeny of field-captured individuals of *Ae. aegypti* from the Lower Keys (Fig 4). This may suggest that wild type populations of this anthropophagic mosquito possess higher vector competence than the Orlando strain, which has been propagated in the laboratory for numerous generations, and may not be representative of field strains [38]. For example, for the 5-day IP, 14-day EIP cohort, 87.5% of TRVL9760-infected females and 62.5% of 240023-infected females were found to have disseminated infections (Table 1). These rates of virus dissemination are substantially higher than dissemination rates reported in previous studies with this same species [13] and higher than the Orlando strain in the current study (19.6% overall). Nonetheless, only one female (out of 40 total samples from individuals with confirmed viral dissemination) from the recently colonized Lower Keys strain of *Ae. aegypti* was found to have OROV RNA in her saliva (TRVL9760, 5-day IP, 14-day EIP), indicating a low overall potential for OROV transmission. This observation was further supported by our quantitative RT-qPCR data. The distribution of Cq values across mosquito species, virus genotypes, and extrinsic incubation periods aligned with the patterns observed in infection, dissemination, and transmission rates. Lower Cq values, which indicate higher viral RNA loads, were most frequently detected in *Ae. aegypti* (Lower Keys and Orlando strains), especially those exposed to the 240023 genotype at earlier time points. In contrast, higher Cq values approaching the assay detection threshold were more common in *Cx. quinquefasciatus* and in mosquitoes exposed to the TRVL9760 genotype. These findings add resolution to within-host viral dynamics and underscore species- and genotype-specific differences in OROV vector competence, but they do not indicate a consistent genotype advantage; instead, mosquito species/strain and timing (IP/EIP) were the dominant drivers of the outcome of measurements of vector competence.

Small changes in the viral genome can have large consequences for transmissibility in vector species. For example, a single amino acid substitution (mutation) in the genome of chikungunya virus was associated with enhanced infectivity for *Ae. albopictus,* which contributed to an epidemic in a region lacking the primary vector, *Ae. aegypti* [21]. Similarly, Venezuelan Equine Encephalitis virus (VEEV) exists as six antigenic subtypes (I through VI) and only subtypes IAB and IC (not II through VI) are associated with epidemics and epizootics attributable to a viremia in equines sufficient for efficient amplification [39]. In the case of OROV, reassortant strains have been documented during recent outbreaks in South America, highlighting that genomic segment diversity may influence virus–vector interactions [19]. Although both genotypes in our study reached comparable PFU titers in Vero cells at the same harvest day (Fig 3), they differ across multiple genomic segments, which could potentially influence early infection processes in mosquitoes. In our experiments, genotype and the length of incubation periods (both IP and EIP) were associated with differences in infection rates across mosquito species and strains, but these effects were inconsistent and did not extend to dissemination or transmission (Table 1). These findings suggest that susceptibility to initial infection is shaped by multiple interacting factors, including viral genotype and environmental factors (particularly at low titers), whereas midgut escape and salivary gland barriers play a more decisive role in regulating whether the virus can disseminate and replicate downstream [40–42].

Despite the widespread occurrence of OROV in the American tropics and the large numbers of human cases that have been documented, robust information on the specific arthropod vectors of OROV in nature is relatively scarce. While several species of blood-feeding Diptera have been found naturally infected with OROV, relatively few published studies have experimentally evaluated the competence of suspected vectors, especially when compared to the extensive body of work on other arboviruses [13,14,43,44]. Our results, and those of other studies indicate that mosquitoes are not likely to transmit OROV to any appreciable level. Notably, the threshold viral titer required to establish oral infection in mosquitoes, often reaching 9.5 log₁₀ SMLD₅₀ per mL, is substantially higher than that required for *C. paraensis* (approximately 5.3 log₁₀ SMLD₅₀ per mL), and typically exceeds the viremia levels observed in human OROV infections, which are generally below 6 log₁₀ PFU/mL [3,8,12,45]. Consequently, females of *Ae. aegypti* and *Cx. quinquefasciatus* feeding on viremic humans in Florida are unlikely to acquire enough virus to overcome midgut and salivary gland barriers, rendering them ineffective vectors of OROV. The consistency of findings across studies despite differences in viral genotypes, detection assays, and experimental designs, strongly supports the conclusion that anthropophagic mosquitoes will not sustain local transmission of OROV in Florida.

The potential role of *Culicoides* in autochthonous transmission of OROV in Florida bears examination. *Culicoides paraensis,* the putative vector of OROV in South America, occurs throughout the eastern USA, where it is primarily associated with forested habitats, due to its larval habitat (damp tree holes) [44]. While *C. paraensis* can occasionally reach densities sufficient to become a biting pest in the U.S. [46], biting rates of this species rarely exceed 10 per day [47]. This is in stark contrast to Amazonas and Pará States in Brazil, where *C. paraensis* utilizes the banana stumps and discarded cacao pods for breeding, and biting rates in excess of 1,000 per hour have been observed [8]. The much lower density of *C. paraensis* in the US will likely limit its importance in OROV transmission. This species was recently documented for the first time in Cuba and was found to be present in areas of OROV outbreaks, likely indicating that it transmitted OROV in the Cuban outbreaks [48]. The finding that *Culicoides sonorensis*, a vector of Orbiviruses affecting ungulates in North America, is capable of transmitting OROV [32] further supports the need to evaluate the vector competence of other biting midges that frequently bite humans. *Culicoides sonorensis* prefers large mammals and is not known to frequently bite humans [49].

In summary, our laboratory evaluation of the vector competence of anthropophagic mosquito species in Florida using prototype and emerging strains of OROV indicates that these mosquitoes are unlikely to support substantial local transmission of the virus. Importantly, our results are based on detection of OROV RNA via RT-qPCR, which does not distinguish between infectious and noninfectious viral particles. This methodological difference likely accounts for the higher infection and dissemination rates observed in our study compared to those based on assays for infectious virus. Although we detected OROV RNA in five saliva samples by RT-qPCR, only two contained infectious virus by plaque assay (S5 Table). This likely reflects the much higher sensitivity of RT-qPCR, which can detect RNA from noninfectious or degraded virions, while plaque assays only detect replication-competent virus. The small saliva volumes available and potential loss of infectivity during handling may also contribute to plaque assay negativity. These findings highlight that RT-qPCR detection does not always indicate transmission potential. To provide a fuller picture, we present both total infection, dissemination, and transmission rates (out of all exposed mosquitoes; S1 Table) and conditional rates (measured only among individuals that progressed to the preceding stage; Table 1). For example, transmission was calculated as the proportion of mosquitoes with virus-positive saliva among those with confirmed disseminated infections. Conditional rates do not correct for potential overestimation by RT-qPCR but instead highlight the efficiency and barriers at each stage of infection within mosquitoes. Even under permissive laboratory conditions, the low proportion of mosquitoes with detectable viral RNA in saliva, combined with the lack of confirmation of infectious virus in most samples, suggests that *Ae. aegypti* and *Cx. quinquefasciatus* are inefficient vectors for OROV. Future studies should prioritize vector competence assessments using infectious virus detection methods such as plaque assay or TCID_50_, examine additional potential vectors such as anthropophagic *Culicoides* spp., and evaluate the potential for vertical and venereal transmission in candidate vector populations.

## Supporting information

S1 Table The mean infection, dissemination, and transmission rates (total rates) for *Culex quinquefasciatus* (Vero Beach strain, 2015) and *Aedes aegypti* (Orlando strain, 1952; Lower Keys strain, 2024) were assessed following exposure to two Oropouche virus genotypes (TRVL9760 and 240023), using virus suspensions incubated for 5 or 7 days in cell culture (incubation period in cell culture, IP). The Lower Keys strain represents F₁ progeny derived from field-collected *Ae. aegypti* in 2024. Rates were evaluated at 7-, 14-, and 21-day extrinsic incubation periods (EIP). Infection, dissemination, and transmission rates were calculated as follows: infection = number of mosquitoes with OROV-positive bodies ÷ total number tested; dissemination = number of mosquitoes with OROV-positive legs or wings ÷ total number tested; transmission = number of mosquitoes with OROV-positive saliva ÷ total number tested. All samples were tested for OROV RNA using RT-qPCR, and samples with Cq values ≤38 were considered positive. A dash (–) indicates that samples were not collected.
(DOCX)

S2 Table Logistic regression analyses were conducted to evaluate the effects of species or strain, OROV genotype (TRVL9760 and 240023), incubation period in cell culture (IP: 5 and 7 days), extrinsic incubation period (EIP: 7, 14, and 21 days), and their interactions on OROV positivity in infection (head, thorax, and abdomen), dissemination (legs and wings), and transmission (saliva). Values in boldface indicate that the effect was significant (*p* < 0.05).
(DOCX)

S3 Table Mean (SD) Cq values of Oropouche virus (OROV) RNA detected in mosquito tissues across virus genotypes, mosquito species, and experimental conditions. Cq values represent the mean quantification cycle (Cq) ± standard deviation (SD) for each sample type (infection, dissemination, transmission) in *Culex quinquefasciatus* and *Aedes aegypti* (Orlando and Lower Keys strains) exposed to two OROV genotypes (TRVL9760 and 240023). Time points correspond to extrinsic incubation periods (EIP) following exposure to virus incubated in cell culture for either 5 or 7 days (IP). Samples with Cq values ≤38 were considered positive, based on CDC interpretation criteria for OROV RT-qPCR using the SuperScript III platform. Values near this threshold should be interpreted with caution, as they may reflect low-titer viral RNA near the assay’s detection limit. A dash (–) indicates that samples were not collected. Cq values without accompanying SD reflect single detections. Empty cells indicate that no positive samples were available for that condition.
(DOCX)

S4 Table (1) Infection, (2) dissemination, and (3) transmission rates and Wilson score 95% confidence intervals for *Culex quinquefasciatus* (Vero Beach strain, 2015) and *Aedes aegypti* (Orlando strain, 1952; Lower Keys strain, 2024) exposed to two Oropouche virus (OROV) genotypes (TRVL9760 and 240023) under different incubation periods in cell culture (IP) and extrinsic incubation periods (EIP). “Positive (N)” refers to the number of mosquitoes with RT-qPCR–positive bodies, and “Total (N)” refers to the total number of mosquitoes tested. “Positivity (%)” indicates the proportion positive, with lower and upper 95% confidence limits calculated using the Wilson score method. RT-qPCR positivity was defined as a Cq value ≤38.
(DOCX)

S5 Table Detection of Oropouche virus (OROV) in mosquito saliva by RT-qPCR and plaque assay. IP = incubation period of virus in cell culture before mosquito exposure; EIP = extrinsic incubation period in mosquitoes. Cq values represent viral RNA detected by RT-qPCR; PFU/mL indicates infectious virus detected by plaque assay. ND = not detectable. RT-qPCR positivity was defined as a Cq value ≤38.
(DOCX)

S1 Fig Oropouche standard curves for OROV (A) TRVL9760 and (B) 240023. Viral titer was determined by a standard plaque assay using Vero cell monolayers in 12-well plates (3 replicates). The mean Cq value of 10-fold serial dilutions of OROV RNA as determined by the RT-qPCR assay was plotted.
(TIF)
