# Supplementary material for: Anthropophagic Florida mosquito species are poor vectors of prototype and emerging strains of oropouche virus
Source: PLoS Negl Trop Dis. 2025 Dec 1;19(12):e0013755. doi: 10.1371/journal.pntd.0013755 (PMC12680353; doi:10.1371/journal.pntd.0013755)
Supplement: S1 Table — The Lower Keys strain represents F₁ progeny derived from field-collected Ae. aegypti in 2024. Rates were evaluated at 7-, 14-, and 21-day extrinsic incubation periods (EIP). Infection, dissemination, and transmission rates were calculated as follows: infection = number of mosquitoes with OROV-positive bodies ÷ total number tested; dissemination = number of mosquitoes with OROV-positive legs or wings ÷ total number tested; transmission = number of mosquitoes with OROV-positive saliva ÷ total number tested. All samples were tested for OROV RNA using RT-qPCR, and samples with Cq values ≤38 were considered positive. A dash (–) indicates that samples were not collected. (DOCX) [file pntd.0013755.s001.docx]

**S1 Table.** The mean infection, dissemination, and transmission rates (total rates) for *Culex quinquefasciatus* (Vero Beach strain, 2015) and *Aedes aegypti* (Orlando strain, 1952; Lower Keys strain, 2024) were assessed following exposure to two Oropouche virus genotypes (TRVL9760 and 240023), using virus suspensions incubated for 5 or 7 days in cell culture (intrinsic incubation period, IP). The Lower Keys strain represents F₁ progeny derived from field-collected *Ae. aegypti* in 2024. Rates were evaluated at 7-, 14-, and 21-day extrinsic incubation periods (EIP). Infection, dissemination, and transmission rates were calculated as follows: infection = number of mosquitoes with OROV-positive bodies ÷ total number tested; dissemination = number of mosquitoes with OROV-positive legs or wings ÷ total number tested; transmission = number of mosquitoes with OROV-positive saliva ÷ total number tested. All samples were tested for OROV RNA using RT-qPCR, and samples with Cq values ≤38 were considered positive. A dash (–) indicates that samples were not collected.

| **OROV Genotype** | **Mosquito Species** | **Strain** | **Sample Type** | **5**  **IP** | | | **7**  **IP** | | |
| --- | --- | --- | --- | --- | --- | --- | --- | --- | --- |
|  |  |  |  | **7  EIP** | **14  EIP** | **21  EIP** | **7  EIP** | **14  EIP** | **21  EIP** |
| TRVL9760 | *Cx. quinquefasciatus* | Vero Beach | Infection  (% [n/N]) | 26.1%  (12/46) | 30.4%  (14/46) | 15.2%  (7/46) | 17.4%  (8/46) | 10.9%  (5/46) | 14.6%  (7/48) |
|  |  |  | Dissemination  (% [n/N]) | 19.6%  (9/46) | 2.2%  (1/46) | 0.0%  (0/46) | 6.5%  (3/46) | 0.0%  (0/46) | 4.2%  (2/48) |
|  |  |  | Transmission  (% [n/N]) | 0.0%  (0/46) | 0.0%  (0/46) | 0.0%  (0/46) | 0.0%  (0/46) | 0.0%  (0/46) | 0.0%  (0/48) |
|  | *Ae. aegypti* | Orlando | Infection  (% [n/N]) | 2.2%  (10/46) | 60.9%  (28/46) | 19.6%  (9/46) | 40.0%  (16/40) | 4.3%  (2/46) | 4.2%  (2/48) |
|  |  |  | Dissemination  (% [n/N]) | 0.0%  (0/46) | 8.7%  (4/46) | 0.0%  (0/46) | 10.0%  (4/40) | 0.0%  (0/46) | 2.1%  (1/48) |
|  |  |  | Transmission  (% [n/N]) | 0.0%  (0/46) | 0.0%  (0/46) | 0.0%  (0/46) | 2.5%  (1/40) | 0.0%  (0/46) | 0.0%  (0/48) |
|  | *Ae. aegypti* | Lower Keys | Infection  (% [n/N]) | - | 66.7%  (8/12) | - | - | 52.9%  (9/17) | - |
|  |  |  | Dissemination  (% [n/N]) | - | 58.3%  (7/12) | - | - | 29.4%  (5/17) | - |
|  |  |  | Transmission  (% [n/N]) | - | 8.3%  (1/12) | - | - | 0.0%  (0/17) | - |
| 240023 | *Cx. quinquefasciatus* | Vero Beach | Infection  (% [n/N]) | 19.6%  (9/46) | 23.9%  (11/46) | 80.4%  (37/46) | 26.1%  (12/46) | 15.2%  (7/46) | 10.9%  (5/46) |
|  |  |  | Dissemination  (% [n/N]) | 13.0%  (6/46) | 0.0%  (0/46) | 13.0%  (6/46) | 8.7%  (4/46) | 0.0%  (0/46) | 2.2%  (1/46) |
|  |  |  | Transmission  (% [n/N]) | 0.0%  (0/46) | 2.2%  (1/46) | 0.0%  (0/46) | 0.0%  (0/46) | 0.0%  (0/46) | 0.0%  (0/46) |
|  | *Ae. aegypti* | Orlando | Infection  (% [n/N]) | 60.9%  (28/46) | 21.7%  (10/46) | 19.6%  (9/46) | 34.8%  (16/46) | 12.5%  (6/48) | 63.0%  (29/46) |
|  |  |  | Dissemination  (% [n/N]) | 34.8%  (16/46) | 8.7%  (4/46) | 0.0%  (0/46) | 4.3%  (2/46) | 2.1%  (1/48) | 13.0%  (6/46) |
|  |  |  | Transmission  (% [n/N]) | 2.2%  (0/46) | 0.0%  (0/46) | 0.0%  (0/46) | 2.2%  (1/46) | 0.0%  (0/48) | 0.0%  (0/46) |
|  | *Ae. aegypti* | Lower Keys | Infection  (% [n/N]) | - | 37.0%  (17/46) | - | - | 35.0%  (7/20) | - |
|  |  |  | Dissemination  (% [n/N]) | - | 21.7%  (10/46) | - | - | 5.0%  (1/20) | - |
|  |  |  | Transmission  (% [n/N]) | - | 0.0%  (0/46) | - | - | 0.0%  (0/20) | - |
