# Supplementary material for: Anthropophagic Florida mosquito species are poor vectors of prototype and emerging strains of oropouche virus
Source: PLoS Negl Trop Dis. 2025 Dec 1;19(12):e0013755. doi: 10.1371/journal.pntd.0013755 (PMC12680353; doi:10.1371/journal.pntd.0013755)
Supplement: S2 Table — Values in boldface indicate that the effect was significant (p < 0.05). (DOCX) [file pntd.0013755.s002.docx]

**S2 Table.** Logistic regression analyses were conducted to evaluate the effects of species or strain, OROV genotype (TRVL9760 and 240023), incubation period in cell culture (IP: 5 and 7 days), extrinsic incubation period (EIP: 7, 14, and 21 days), and their interactions on OROV positivity in infection (head, thorax, and abdomen), dissemination (legs and wings), and transmission (saliva). Values in boldface indicate that the effect was significant (*p* < 0.05).

| **Type** | **Factor/interaction** | ***df*** | **L-R ChiSq.** | ***p*** |
| --- | --- | --- | --- | --- |
| **Infection** | **Species/Strain** | 2 | 16.2187396 | **0.0003** |
|  | **OROV Genotype** | 1 | 9.70566576 | **0.0018** |
|  | **IP** | 1 | 19.4908896 | **<.0001** |
|  | **EIP** | 2 | 6.84114264 | **0.0327** |
|  | **Species/Strain*OROV Genotype** | 2 | 6.99353288 | **0.0303** |
|  | **Species/Strain*IP** | 2 | 24.894253 | **<.0001** |
|  | **Species/Strain*EIP** | 4 | 38.6764457 | **<.0001** |
|  | OROV Genotype*IP | 1 | 0.05174997 | 0.82 |
|  | **OROV Genotype*EIP** | 2 | 28.9909568 | **<.0001** |
|  | **IP*EIP** | 2 | 23.0964545 | **<.0001** |
|  | **Species/Strain*OROV Genotype*IP** | 2 | 9.41988268 | **0.009** |
|  | **Species/Strain*OROV Genotype*EIP** | 4 | 23.4318968 | **0.0001** |
|  | **Species/Strain*IP*EIP** | 4 | 29.4845356 | **<.0001** |
|  | **OROV Genotype*IP*EIP** | 2 | 8.03621743 | **0.018** |
|  | **Species/Strain*OROV Genotype*IP*EIP** | 4 | 59.434589 | **<.0001** |
| **Dissemination** | **Species/Strain** | 2 | 29.5227198 | **<.0001** |
|  | OROV Genotype | 1 | 0.15836593 | 0.6907 |
|  | **IP** | 1 | 5.36914505 | **0.0205** |
|  | **EIP** | 2 | 29.0975405 | **<.0001** |
|  | Species/Strain*OROV Genotype | 2 | 5.72284739 | 0.0572 |
|  | Species/Strain*IP | 2 | 3.36141335 | 0.1862 |
|  | **Species/Strain*EIP** | 4 | 60.0943484 | **<.0001** |
|  | **OROV Genotype*IP** | 1 | 7.3758125 | **0.0066** |
|  | OROV Genotype*EIP | 2 | 1.42029402 | 0.4916 |
|  | **IP*EIP** | 2 | 20.6074079 | **<.0001** |
|  | Species/Strain*OROV Genotype*IP | 2 | 4.67171215 | 0.0967 |
|  | Species/Strain*OROV Genotype*EIP | 4 | 5.01007666 | 0.2863 |
|  | **Species/Strain*IP*EIP** | 4 | 22.9615324 | **0.0001** |
|  | OROV Genotype*IP*EIP | 2 | 0.57470806 | 0.7502 |
|  | Species/Species/Strain*OROV Genotype*IP*EIP | 4 | 7.06614835 | 0.1324 |
| **Transmission** | Species/Strain | 2 | 0.57978937 | 0.7483 |
|  | OROV Genotype | 1 | 0.04654116 | 0.8292 |
|  | IP | 1 | 3.50E-05 | 0.9953 |
|  | EIP | 2 | 3.76527859 | 0.1522 |
|  | Species/Strain*OROV Genotype | 2 | 3.95555577 | 0.1384 |
|  | Species/Strain*IP | 2 | 5.94E-06 | 1 |
|  | Species/Strain*EIP | 4 | 5.63327425 | 0.2283 |
|  | OROV Genotype*IP | 1 | 0.51263549 | 0.474 |
|  | OROV Genotype*EIP | 2 | 0.00001404 | 1 |
|  | IP*EIP | 2 | 0.00014646 | 0.9999 |
|  | **Species/Strain*OROV Genotype*IP** | 2 | 8.81361219 | **0.0122** |
|  | **Species/Strain*OROV Genotype*EIP** | 4 | 9.80952421 | **0.0438** |
|  | Species/Strain*IP*EIP | 4 | 8.52778934 | 0.074 |
|  | OROV Genotype*IP*EIP | 2 | 1.86223851 | 0.3941 |
|  | Species/Strain*OROV Genotype*IP*EIP | 4 | 4.33379167 | 0.3627 |
