# Supplementary material for: Anthropophagic Florida mosquito species are poor vectors of prototype and emerging strains of oropouche virus
Source: PLoS Negl Trop Dis. 2025 Dec 1;19(12):e0013755. doi: 10.1371/journal.pntd.0013755 (PMC12680353; doi:10.1371/journal.pntd.0013755)
Supplement: S3 Table — Cq values represent the mean quantification cycle (Cq) ± standard deviation (SD) for each sample type (infection, dissemination, transmission) in Culex quinquefasciatus and Aedes aegypti (Orlando and Lower Keys strains) exposed to two OROV genotypes (TRVL9760 and 240023). Time points correspond to extrinsic incubation periods (EIP) following exposure to virus incubated in cell culture for either 5 or 7 days (IP). Samples with Cq values ≤38 were considered positive, based on CDC interpretation criteria for OROV RT-qPCR using the SuperScript III platform. Values near this threshold should be interpreted with caution, as they may reflect low-titer viral RNA near the assay’s detection limit. A dash (–) indicates that samples were not collected. Cq values without accompanying SD reflect single detections. Empty cells indicate that no positive samples were available for that condition. (DOCX) [file pntd.0013755.s003.docx]

**S3 Table.** Mean (SD) Cq values of Oropouche virus (OROV) RNA detected in mosquito tissues across virus genotypes, mosquito species, and experimental conditions. Cq values represent the mean quantification cycle (Cq) ± standard deviation (SD) for each sample type (infection, dissemination, transmission) in *Culex quinquefasciatus* and *Aedes aegypti* (Orlando and Lower Keys strains) exposed to two OROV genotypes (TRVL9760 and 240023). Time points correspond to extrinsic incubation periods (EIP) following exposure to **blood meals prepared from virus suspensions incubated in cell culture for 5 days (higher OROV titers) or 7 days (lower OROV titers), as indicated in the table.** Samples with Cq values ≤38 were considered positive, based on CDC interpretation criteria for OROV RT-qPCR using the SuperScript III platform. Values near this threshold should be interpreted with caution, as they may reflect low-titer viral RNA near the assay’s detection limit. A dash (–) indicates that samples were not collected. Cq values without accompanying SD reflect single detections. Numbers in brackets (n) indicate the number of positive mosquitoes contributing to each mean ± SD. Empty cells indicate that no positive samples were available for that condition.

| **OROV Genotype** | **Mosquito Species** | **Strain** | **Sample Type** | **5 IP (Higher OROV titer)** | | | **7 IP**  **(Lower OROV titer)** | | |
| --- | --- | --- | --- | --- | --- | --- | --- | --- | --- |
|  |  |  |  | **7  EIP** | **14  EIP** | **21  EIP** | **7  EIP** | **14  EIP** | **21  EIP** |
| TRVL9760 | *Cx. quinquefasciatus* | Vero Beach | Infection | 32.2  (3.7) [n=12] | 33.6  (2.0)  [n=14] | 35.2  (1.5)  [n=7] | 33.8  (3.3)  [n=8] | 35.5  (2.2)  [n=5] | 34.0  (6.1)  [n=7] |
|  |  |  | Dissemination | 33.7  (1.4)  [n=9] | 36.7  [n=1] |  | 36.5  (1.2)  [n=3] |  | 34.9  (2.5)  [n=2] |
|  |  |  | Transmission |  |  |  |  |  |  |
|  | *Ae. aegypti* | Orlando | Infection | 31.9  (5.1)  [n=10] | 32.3  (4.3)  [n=28] | 29.9  (4.9)  [n=9] | 31.4  (3.7) [n=16] | 32.4  (4.3)  [n=2] | 35.9  (1.1)  [n=2] |
|  |  |  | Dissemination |  | 35.9  (1.7)  [n=4] |  | 37.4  (0.2)  [n=4] |  | 37.5  [n=1] |
|  |  |  | Transmission |  |  |  | 36.9  [n=1] |  |  |
|  | *Ae. aegypti* | Lower Keys | Infection | - | 32.0  (6.7)  [n=12] | - | - | 32.2  (3.2)  [n=9] | - |
|  |  |  | Dissemination | - | 33.5  (6.8)  [n=7] | - | - | 35.2  (1.9)  [n=5] | - |
|  |  |  | Transmission | - | 27.9  [n=1] | - | - |  | - |
| 240023 | *Cx. quinquefasciatus* | Vero Beach | Infection | 33.7  (5.9)  [n=9] | 34.2  (3.2)  [n=11] | 34.5  (3.9)  [n=37] | 32.2  (4.2)  [n=12] | 32.8  (1.9)  [n=7] | 31.1  (7.2)  [n=5] |
|  |  |  | Dissemination | 34.7  (2.0)  [n=6] |  | 35.2  (2.0)  [n=6] | 31.8  (7.1)  [n=4] |  | 37.7  [n=1] |
|  |  |  | Transmission |  | 37.7  [n=1] |  |  |  |  |
|  | *Ae. aegypti* | Orlando | Infection | 32.4  (4.9)  [n=28] | 33.0  (6.2)  [n=10] | 34.7  (1.9)  [n=9] | 30.6  (4.6)  [n=16] | 31.9  (5.8)  [n=6] | 32.9  (4.4)  [n=29] |
|  |  |  | Dissemination | 33.0  (4.1)  [n=16] | 35.1  (1.4)  [n=4] |  | 29.9  (10.2)  [n=2] | 36.5  [n=1] | 35.0  (3.2)  [n=6] |
|  |  |  | Transmission | 35.6  [n=1] |  |  | 37.4  [n=1] |  |  |
|  | *Ae. aegypti* | Lower Keys | Infection | - | 30.8  (5.0)  [n=17] | - | - | 29.5  (4.4)  [n=7] | - |
|  |  |  | Dissemination | - | 34.4  (0.8)  [n=10] | - | - | 37.6  [n=1] | - |
|  |  |  | Transmission | - |  | - | - |  | - |
