# Supplementary material for: Anthropophagic Florida mosquito species are poor vectors of prototype and emerging strains of oropouche virus
Source: PLoS Negl Trop Dis. 2025 Dec 1;19(12):e0013755. doi: 10.1371/journal.pntd.0013755 (PMC12680353; doi:10.1371/journal.pntd.0013755)
Supplement: S4 Table — “Positive (N)” refers to the number of mosquitoes with RT-qPCR–positive bodies, and “Total (N)” refers to the total number of mosquitoes tested. “Positivity (%)” indicates the proportion positive, with lower and upper 95% confidence limits calculated using the Wilson score method. RT-qPCR positivity was defined as a Cq value ≤38. (DOCX) [file pntd.0013755.s004.docx]

**S4 Table.** (1) Infection, (2) dissemination, and (3) transmission rates and Wilson score 95% confidence intervals for Culex quinquefasciatus (Vero Beach strain, 2015) and Aedes aegypti (Orlando strain, 1952; Lower Keys strain, 2024) exposed to two Oropouche virus (OROV) genotypes (TRVL9760 and 240023) under different incubation periods in cell culture (IP) and extrinsic incubation periods (EIP). "Positive (N)" refers to the number of mosquitoes with RT-qPCR–positive bodies, and "Total (N)" refers to the total number of mosquitoes tested. "Positivity (%)" indicates the proportion positive, with lower and upper 95% confidence limits calculated using the Wilson score method. RT-qPCR positivity was defined as a Cq value ≤38.

**(1) Infection rates**

| **Strain** | **Genotype** | **Infection type** | **IP** | **EIP** | **Positive (N)** | **Total (N)** | **Positivity (%)** | **95% CI Lower** | **95% CI Upper** |
| --- | --- | --- | --- | --- | --- | --- | --- | --- | --- |
| Lower keys | 240023 | Infection | 5 | 14 | 17 | 46 | 36.96 | 24.52 | 51.40 |
| Lower keys | 240023 | Infection | 7 | 14 | 7 | 20 | 35.00 | 18.12 | 56.71 |
| Lower keys | TRVL9760 | Infection | 5 | 14 | 8 | 12 | 66.67 | 39.06 | 86.19 |
| Lower keys | TRVL9760 | Infection | 7 | 14 | 9 | 17 | 52.94 | 30.96 | 73.83 |
| Orlando | 240023 | Infection | 5 | 7 | 28 | 46 | 60.87 | 46.46 | 73.61 |
| Orlando | 240023 | Infection | 5 | 14 | 10 | 46 | 21.74 | 12.26 | 35.57 |
| Orlando | 240023 | Infection | 5 | 21 | 9 | 46 | 19.57 | 10.65 | 33.17 |
| Orlando | 240023 | Infection | 7 | 7 | 16 | 46 | 34.78 | 22.68 | 49.23 |
| Orlando | 240023 | Infection | 7 | 14 | 6 | 48 | 12.50 | 5.86 | 24.70 |
| Orlando | 240023 | Infection | 7 | 21 | 29 | 46 | 63.04 | 48.60 | 75.48 |
| Orlando | TRVL9760 | Infection | 5 | 7 | 10 | 46 | 21.74 | 12.26 | 35.57 |
| Orlando | TRVL9760 | Infection | 5 | 14 | 28 | 46 | 60.87 | 46.46 | 73.61 |
| Orlando | TRVL9760 | Infection | 5 | 21 | 9 | 46 | 19.57 | 10.65 | 33.17 |
| Orlando | TRVL9760 | Infection | 7 | 7 | 16 | 40 | 40.00 | 26.35 | 55.40 |
| Orlando | TRVL9760 | Infection | 7 | 14 | 2 | 46 | 4.35 | 1.20 | 14.53 |
| Orlando | TRVL9760 | Infection | 7 | 21 | 2 | 48 | 4.17 | 1.15 | 13.98 |
| Vero beach | 240023 | Infection | 5 | 7 | 9 | 46 | 19.57 | 10.65 | 33.17 |
| Vero beach | 240023 | Infection | 5 | 14 | 11 | 46 | 23.91 | 13.91 | 37.94 |
| Vero beach | 240023 | Infection | 5 | 21 | 37 | 46 | 80.43 | 66.83 | 89.35 |
| Vero beach | 240023 | Infection | 7 | 7 | 12 | 46 | 26.09 | 15.60 | 40.26 |
| Vero beach | 240023 | Infection | 7 | 14 | 7 | 46 | 15.22 | 7.57 | 28.22 |
| Vero beach | 240023 | Infection | 7 | 21 | 5 | 46 | 10.87 | 4.73 | 23.04 |
| Vero beach | TRVL9760 | Infection | 5 | 7 | 12 | 46 | 26.09 | 15.60 | 40.26 |
| Vero beach | TRVL9760 | Infection | 5 | 14 | 14 | 46 | 30.43 | 19.08 | 44.81 |
| Vero beach | TRVL9760 | Infection | 5 | 21 | 7 | 46 | 15.22 | 7.57 | 28.22 |
| Vero beach | TRVL9760 | Infection | 7 | 7 | 8 | 46 | 17.39 | 9.09 | 30.72 |
| Vero beach | TRVL9760 | Infection | 7 | 14 | 5 | 46 | 10.87 | 4.73 | 23.04 |
| Vero beach | TRVL9760 | Infection | 7 | 21 | 7 | 48 | 14.58 | 7.25 | 27.17 |

**(2) Dissemination rates**

| **Strain** | **Genotype** | **Infection type** | **IP** | **EIP** | **Positive (N)** | **Total (N)** | **Positivity (%)** | **95% CI Lower** | **95% CI Upper** |
| --- | --- | --- | --- | --- | --- | --- | --- | --- | --- |
| Lower keys | 240023 | Dissemination | 5 | 14 | 10 | 16 | 62.50 | 38.64 | 81.52 |
| Lower keys | 240023 | Dissemination | 7 | 14 | 1 | 7 | 14.29 | 2.57 | 51.31 |
| Lower keys | TRVL9760 | Dissemination | 5 | 14 | 7 | 8 | 87.50 | 52.91 | 97.76 |
| Lower keys | TRVL9760 | Dissemination | 7 | 14 | 5 | 9 | 55.56 | 26.67 | 81.12 |
| Orlando | 240023 | Dissemination | 5 | 7 | 16 | 28 | 57.14 | 39.07 | 73.49 |
| Orlando | 240023 | Dissemination | 5 | 14 | 4 | 10 | 40.00 | 16.82 | 68.73 |
| Orlando | 240023 | Dissemination | 5 | 21 | 0 | 9 | 0.00 | 0.00 | 29.91 |
| Orlando | 240023 | Dissemination | 7 | 7 | 2 | 16 | 12.50 | 3.50 | 36.02 |
| Orlando | 240023 | Dissemination | 7 | 14 | 1 | 6 | 16.67 | 3.01 | 56.35 |
| Orlando | 240023 | Dissemination | 7 | 21 | 6 | 29 | 20.69 | 9.85 | 38.39 |
| Orlando | TRVL9760 | Dissemination | 5 | 7 | 0 | 10 | 0.00 | 0.00 | 27.75 |
| Orlando | TRVL9760 | Dissemination | 5 | 14 | 4 | 28 | 14.29 | 5.70 | 31.49 |
| Orlando | TRVL9760 | Dissemination | 5 | 21 | 0 | 9 | 0.00 | 0.00 | 29.91 |
| Orlando | TRVL9760 | Dissemination | 7 | 7 | 4 | 16 | 25.00 | 10.18 | 49.50 |
| Orlando | TRVL9760 | Dissemination | 7 | 14 | 0 | 2 | 0.00 | 0.00 | 65.76 |
| Orlando | TRVL9760 | Dissemination | 7 | 21 | 1 | 2 | 50.00 | 9.45 | 90.55 |
| Vero beach | 240023 | Dissemination | 5 | 7 | 6 | 9 | 66.67 | 35.42 | 87.94 |
| Vero beach | 240023 | Dissemination | 5 | 14 | 0 | 11 | 0.00 | 0.00 | 25.88 |
| Vero beach | 240023 | Dissemination | 5 | 21 | 6 | 37 | 16.22 | 7.65 | 31.14 |
| Vero beach | 240023 | Dissemination | 7 | 7 | 4 | 12 | 33.33 | 13.81 | 60.94 |
| Vero beach | 240023 | Dissemination | 7 | 14 | 0 | 7 | 0.00 | 0.00 | 35.43 |
| Vero beach | 240023 | Dissemination | 7 | 21 | 1 | 5 | 20.00 | 3.62 | 62.45 |
| Vero beach | TRVL9760 | Dissemination | 5 | 7 | 9 | 12 | 75.00 | 46.77 | 91.11 |
| Vero beach | TRVL9760 | Dissemination | 5 | 14 | 1 | 14 | 7.14 | 1.27 | 31.47 |
| Vero beach | TRVL9760 | Dissemination | 5 | 21 | 0 | 7 | 0.00 | 0.00 | 35.43 |
| Vero beach | TRVL9760 | Dissemination | 7 | 7 | 3 | 8 | 37.50 | 13.68 | 69.43 |
| Vero beach | TRVL9760 | Dissemination | 7 | 14 | 0 | 5 | 0.00 | 0.00 | 43.45 |
| Vero beach | TRVL9760 | Dissemination | 7 | 21 | 2 | 7 | 28.57 | 8.22 | 64.11 |

**(3) Transmission rates**

| **Strain** | **Genotype** | **Infection type** | **IP** | **EIP** | **Positive (N)** | **Total (N)** | **Positivity (%)** | **95% CI Lower** | **95% CI Upper** |
| --- | --- | --- | --- | --- | --- | --- | --- | --- | --- |
| Lower keys | 240023 | Transmission | 5 | 14 | 16 | 0 | 0.00 | 0.00 | 19.36 |
| Lower keys | 240023 | Transmission | 7 | 14 | 7 | 0 | 0.00 | 0.00 | 35.43 |
| Lower keys | TRVL9760 | Transmission | 5 | 14 | 8 | 1 | 12.50 | 2.24 | 47.09 |
| Lower keys | TRVL9760 | Transmission | 7 | 14 | 9 | 0 | 0.00 | 0.00 | 29.91 |
| Orlando | 240023 | Transmission | 5 | 7 | 28 | 1 | 3.57 | 0.63 | 17.71 |
| Orlando | 240023 | Transmission | 5 | 14 | 10 | 0 | 0.00 | 0.00 | 27.75 |
| Orlando | 240023 | Transmission | 5 | 21 | 9 | 0 | 0.00 | 0.00 | 29.91 |
| Orlando | 240023 | Transmission | 7 | 7 | 16 | 1 | 6.25 | 1.11 | 28.33 |
| Orlando | 240023 | Transmission | 7 | 14 | 6 | 0 | 0.00 | 0.00 | 39.03 |
| Orlando | 240023 | Transmission | 7 | 21 | 29 | 0 | 0.00 | 0.00 | 11.70 |
| Orlando | TRVL9760 | Transmission | 5 | 7 | 10 | 0 | 0.00 | 0.00 | 27.75 |
| Orlando | TRVL9760 | Transmission | 5 | 14 | 28 | 0 | 0.00 | 0.00 | 12.06 |
| Orlando | TRVL9760 | Transmission | 5 | 21 | 9 | 0 | 0.00 | 0.00 | 29.91 |
| Orlando | TRVL9760 | Transmission | 7 | 7 | 16 | 1 | 6.25 | 1.11 | 28.33 |
| Orlando | TRVL9760 | Transmission | 7 | 14 | 2 | 0 | 0.00 | 0.00 | 65.76 |
| Orlando | TRVL9760 | Transmission | 7 | 21 | 2 | 0 | 0.00 | 0.00 | 65.76 |
| Vero beach | 240023 | Transmission | 5 | 7 | 9 | 0 | 0.00 | 0.00 | 29.91 |
| Vero beach | 240023 | Transmission | 5 | 14 | 11 | 1 | 9.09 | 1.62 | 37.74 |
| Vero beach | 240023 | Transmission | 5 | 21 | 37 | 0 | 0.00 | 0.00 | 9.41 |
| Vero beach | 240023 | Transmission | 7 | 7 | 12 | 0 | 0.00 | 0.00 | 24.25 |
| Vero beach | 240023 | Transmission | 7 | 14 | 7 | 0 | 0.00 | 0.00 | 35.43 |
| Vero beach | 240023 | Transmission | 7 | 21 | 5 | 0 | 0.00 | 0.00 | 43.45 |
| Vero beach | TRVL9760 | Transmission | 5 | 7 | 12 | 0 | 0.00 | 0.00 | 24.25 |
| Vero beach | TRVL9760 | Transmission | 5 | 14 | 14 | 0 | 0.00 | 0.00 | 21.53 |
| Vero beach | TRVL9760 | Transmission | 5 | 21 | 7 | 0 | 0.00 | 0.00 | 35.43 |
| Vero beach | TRVL9760 | Transmission | 7 | 7 | 8 | 0 | 0.00 | 0.00 | 32.44 |
| Vero beach | TRVL9760 | Transmission | 7 | 14 | 5 | 0 | 0.00 | 0.00 | 43.45 |
| Vero beach | TRVL9760 | Transmission | 7 | 21 | 7 | 0 | 0.00 | 0.00 | 35.43 |
