# Supplementary material for: Anthropophagic Florida mosquito species are poor vectors of prototype and emerging strains of oropouche virus
Source: PLoS Negl Trop Dis. 2025 Dec 1;19(12):e0013755. doi: 10.1371/journal.pntd.0013755 (PMC12680353; doi:10.1371/journal.pntd.0013755)
Supplement: S5 Table — IP = incubation period of virus in cell culture before mosquito exposure; EIP = extrinsic incubation period in mosquitoes. Cq values represent viral RNA detected by RT-qPCR; PFU/mL indicates infectious virus detected by plaque assay. ND = not detectable. RT-qPCR positivity was defined as a Cq value ≤38. (DOCX) [file pntd.0013755.s005.docx]

**S5 Table.** Detection of Oropouche virus (OROV) in mosquito saliva by RT-qPCR and plaque assay. IP = incubation period of virus in cell culture before mosquito exposure; EIP = extrinsic incubation period in mosquitoes. Cq values represent **viral RNA detected by RT-qPCR;** PFU/mL indicates infectious virus detected by plaque assay. ND = not detectable. **RT-qPCR positivity was defined as a Cq value ≤38.**

| **Species / strain** | **OROV genotype** | **IP (days)** | **EIP (days)** | **% positive (n/N)** | **Cq value**  **(RT-qPCR)** | **PFU/mL** |
| --- | --- | --- | --- | --- | --- | --- |
| *Cx. quinquefasciatus* / Vero Beach | 240023 | 5 | 14 | 9.1% (1/11) | 37.7 | 5 |
| *Ae. aegypti* / Orlando | 240023 | 5 | 7 | 3.6% (1/28) | 35.6 | ND |
| *Ae. aegypti* / Orlando | 240023 | 7 | 7 | 6.3% (1/16) | 38.0 | ND |
| *Ae. aegypti* / Orlando | TRVL9760 | 7 | 7 | 6.3% (1/16) | 36.9 | 25 |
| *Ae. aegypti* / Lower Keys | TRVL9760 | 5 | 14 | 12.5% (1/8) | 27.9 | ND |
