# Supplementary figures and images for: Anthropophagic Florida mosquito species are poor vectors of prototype and emerging strains of oropouche virus
Source: PLoS Negl Trop Dis. 2025 Dec 1;19(12):e0013755. doi: 10.1371/journal.pntd.0013755 (PMC12680353; doi:10.1371/journal.pntd.0013755)

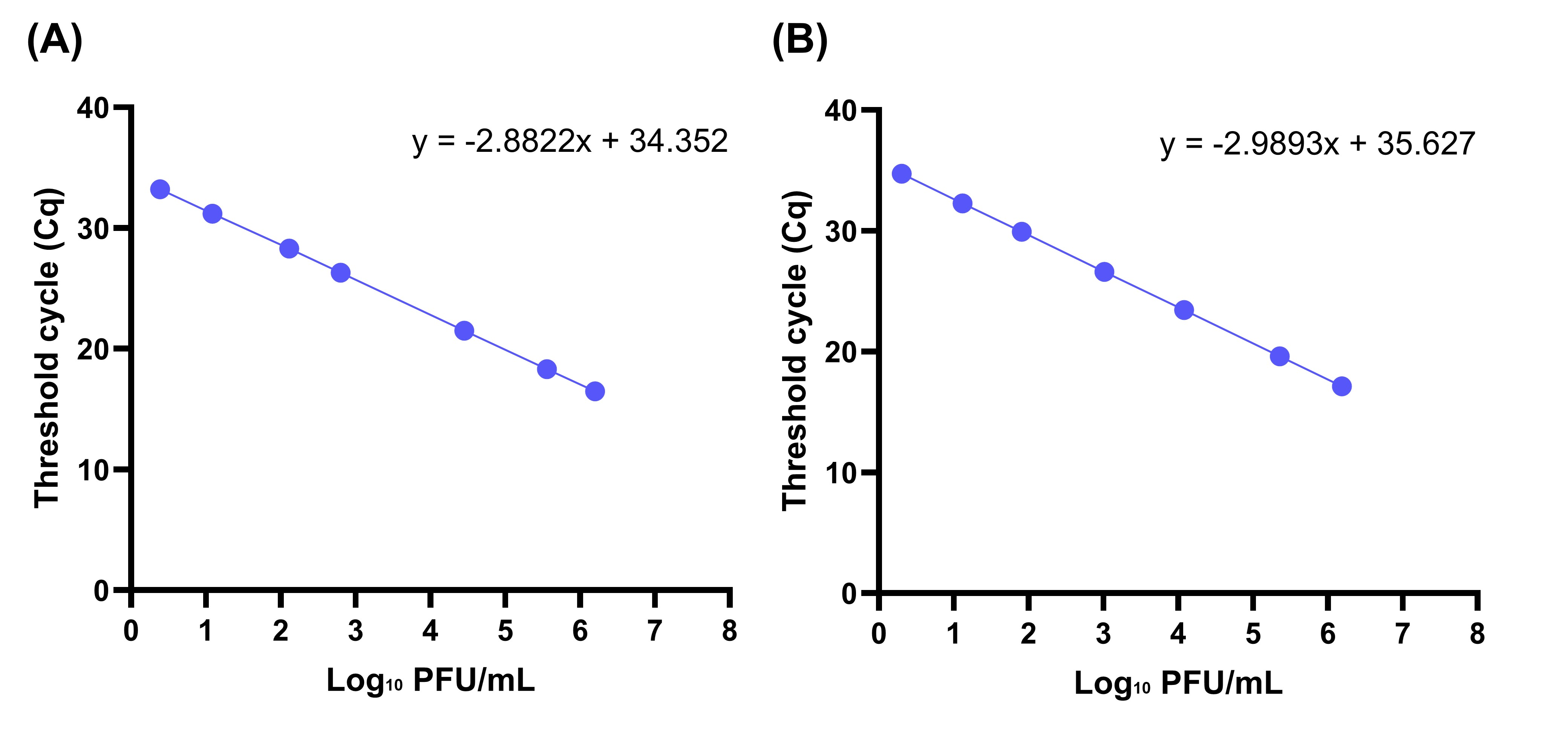

Supplement: S1 Fig — Viral titer was determined by a standard plaque assay using Vero cell monolayers in 12-well plates (3 replicates). The mean Cq value of 10-fold serial dilutions of OROV RNA as determined by the RT-qPCR assay was plotted. (TIF) [file pntd.0013755.s006.tif]
